# Real-life instability in ADHD from young to middle adulthood: a nationwide register-based study of social and occupational problems

**DOI:** 10.1186/s12888-023-04713-z

**Published:** 2023-05-12

**Authors:** Rickard Ahlberg, E. Du Rietz, E. Ahnemark, L. M. Andersson, T. Werner-Kiechle, P. Lichtenstein, H. Larsson, M. Garcia-Argibay

**Affiliations:** 1 School of Medical Sciences, Örebro University, Örebro, 701 82 Sweden; 2 Department of Medical Epidemiology and Biostatistics, Karolinska Institutet, Stockholm, Sweden; 3 Department of Neuropsychiatry, Region Stockholm, Stockholm, Sweden; 4 Takeda Pharma AB, Stockholm, Sweden; 5 Global Medical Affairs, Shire International GmbH, Zug, Switzerland

**Keywords:** Attention/Deficit-Hyperactivity/Disorder, Instability, Adult-ADHD

## Abstract

**Background:**

Studies using self-reports indicate that individuals with ADHD are at increased risk for functional impairments in social and occupational settings, but evidence around real-life instability remains limited. It is furthermore unclear if these functional impairments in ADHD differ across sex and across the adult lifespan.

**Method:**

A longitudinal observational cohort design of 3,448,440 individuals was used to study the associations between ADHD and residential moves, relational instability and job shifting using data from Swedish national registers. Data were stratified on sex and age (18–29 years, 30–39 years, and 40–52 years at start of follow up).

**Results:**

31,081 individuals (17,088 males; 13,993 females) in the total cohort had an ADHD-diagnosis. Individuals with ADHD had an increased incidence rate ratio (IRR) of residential moves (IRR 2.35 [95% CI, 2.32–2.37]), relational instability (IRR = 1.07 [95% CI, 1.06–1.08]) and job shifting (IRR = 1.03 [95% CI, 1.02–1.04]). These associations tended to increase with increasing age. The strongest associations were found in the oldest group (40–52 years at start of follow). Women with ADHD in all three age groups had a higher rate of relational instability compared to men with ADHD.

**Conclusion:**

Both men and women with a diagnosis of ADHD present with an increased risk of real-life instability in different domains and this behavioral pattern was not limited to young adulthood but also existed well into older adulthood. It is therefore important to have a lifespan perspective on ADHD for individuals, relatives, and the health care sector.

**Supplementary Information:**

The online version contains supplementary material available at 10.1186/s12888-023-04713-z.

## Introduction

Attention Deficit/Hyperactivity Disorder (ADHD) is a neurodevelopmental disorder that persists into adulthood for many individuals [1, 2]. The core symptoms of ADHD are a persistent pattern of inattention and/or hyperactivity-impulsivity that interferes with functioning in life. Impulsivity refers to hasty actions that occur in the moment and that can manifest as making important decisions without considerations of long-term consequences (APA, 2013). Inattention problems in adults often leads to difficulties with time management, organizing activities and tasks and following instructions. Emotional dysregulation reflected in emotional impulsivity, negative affect and emotional self-regulation problems has also been considered as a core feature of ADHD [3, 4]. Although instability and impulsivity are characteristics of some other psychiatric disorders, like substance use disorder, borderline personality disorder and antisocial personality disorder there are indications that instability and impulsivity are more prominent features in ADHD [5–8]. These core characteristics of ADHD may manifest in real-life instability in occupational (e.g., job shifting), relational (e.g., divorce), and residential (residential moves) settings. However, a detailed understanding of real-life instability in adult ADHD is currently missing. This is an important limitation because instability in life are important predictors of negative life outcomes [9–11].

The few available observational studies suggest that adults with ADHD present with difficulties in different domains of real-life instability. In a recent narrative review of prospective studies on adult outcomes of children with ADHD followed into adulthood, it was concluded that individuals with ADHD have higher rates of quitting jobs and being fired [12]. Furthermore, a nationwide study in Denmark of welfare consequences for individuals with ADHD found that individuals with ADHD who were in employment had lower income levels than employed individuals without ADHD [13]. Recent narrative reviews of adult ADHD and romantic relationships suggested higher rates of romantic relationship problems for young adults with ADHD compared to those without ADHD [14, 15]. For example, one small study of adults found that inattentive symptoms were associated with greater interest in relational alternatives and less constructive responses to partner’s rude behaviors, whereas hyperactive-impulsive symptoms were associated with negative responses to rude behavior [16]. People with psychiatric conditions, including ADHD, move more often than the general population [17–19]. The increased rates of residential instability in individuals with ADHD could be due to elevated levels of impulsivity and restlessness – an urge to move around between different apartments and different places. ADHD could also be linked to higher rates of residential moves indirectly through higher rates of financial problems [20, 21]. Residential moves in adults are an important outcome because it has a close connection with various health outcomes in children [11, 22]. More research on the association between ADHD and residential instability is needed.

Previous research on ADHD and real-life instability largely consists of studies focused on young adults/individuals < 30 years [12], thus, very little information is available after young adulthood. This is a critical limitation given that the real-life impairments associated with ADHD may change across life. In order to build a more comprehensive lifespan model of real-life instability in ADHD, research on adults beyond young adulthood is needed. Another limitation of the available research on ADHD is that the focus is largely on males, while female presentations having been largely overlooked in both clinical and research settings [15]. The majority of studies on occupational and social instability in ADHD have used small samples, often without sex-specific analyses, and the outcomes are often self-reported through rating scales or by interviews. Self-reported problems in individuals with ADHD, although an important source of information, have been found to be limited in terms of validity [23–25].

Using large national registers from Sweden, the current study examined associations between ADHD and real-life instability over a 14-year period, with a focus on job shifting, relationship instability, and residential moves. The aim was to examine these associations in young and middle adulthood, and also across both males and females. Analyses were adjusted for parent education as previous research has established strong associations between ADHD and parental educational level [26], and because it is an important potential confounder that was fixed at the start of follow up of the individuals in this cohort. In contrast, the individuals own education level is time-varying and may change across follow up. We also included individuals own diagnoses of substance use disorder (SUD), borderline personality disorder (BPD), and criminal convictions until the end of follow up as covariates because previous research has demonstrated that these variables are associated with both ADHD [1, 27, 28] and real-life instability [29, 30].

## Methods

### Data sources and study population

Using the Swedish Total Population Register [31], we identified 3,448,440 individuals born between 1948 and 1982 who were alive and living in Sweden in 2013. All individuals were followed up during 14 years from January 2000 until December 2013. We linked these individuals to several registers by using a unique personal identifier [32]. The Multi Generation Register (MGR) contains information about parents of all individuals born in Sweden from 1932 or registered in the country since 1961 [33]. Using this information, it is possible to identify relatives to the linked individuals. The Integrated Database for Labor Market Research (LISA) contains information about unemployment benefits, disposable income, social welfare payments, civil status, migration, and highest attained education. For occupational data, LISA depends on the Occupation Register and are updated once a year [34]. The National Patient Register (NPR) includes information about somatic and psychiatric diagnoses based on the Swedish version of the International Classification of Diseases (ICD). The National Patient Register (NPR) covers inpatient psychiatric data from 1973 and outpatient psychiatric data from 2001 [35]. The Prescribed Drug Register (PDR) records all prescribed drugs in Sweden from 2005 and onwards [36]. The National crime register contains all convictions in Sweden from 1973 to 2013.

### Exposure

Individuals with ADHD were identified by at least one diagnosis of ADHD in the NPR by using ICD-diagnoses (ICD-9: code 314; ICD-10: code F90) or by at least four dispensations of ADHD medications (The Anatomical Therapeutical Chemical Codes (Amphetamine [N06BA01], Dexamphetamine [N06BA02], Methylphenidate [N06BA04], Lisdexamfetamine [N06BA12]) and non-stimulant medications (Atomoxetine [N06BA09]) in the PDR. Previous research has indicated high specificity for this register-based ADHD definition in Sweden [37, 38]. Furthermore, only physicians specialized in psychiatry/neurology and responsible for ADHD treatment are authorized to prescribe ADHD medication in Sweden, which suggests that prescription of ADHD medication is a valid indicator of ADHD diagnoses.

### Outcome

#### Job shifting

The LISA-register provides information about the extent to which an individual has changed jobs between two consecutive years. Using this information, we calculated a sum score for each individual, reflecting the total number of job changes during the follow-up across 14 years (between January 2000 and December 2013).

***Relationship instability*** was approximated by using the MGR to measure how many children individuals have with different partners during the follow up between January 2000 and December 2013.

#### Residential moves

The LISA-register provides information about residential moves, for each individual, on an annual basis. Using this information, we calculated, for each individual, a sum score reflecting the total number of residential moves during the follow-up across 14 years.

### Controls

The NPR were used to identify psychiatric diagnoses for BPD (F60.3) and SUD using ICD-diagnoses (ICD-8 codes 303 and 304; ICD-9 codes 303–305; ICD-10 codes F10-F19). The validity of the diagnoses of BPD in the NPR has been found to be good in an earlier Swedish register-based study [28]. In line with previous research, the National crime register was used to obtain information about criminal convictions [39]. The LISA register was used to obtain parents highest educational level.

### Statistical analyses

To account for overdispersion, a negative binomial regression using the log link function was used to ascertain the relationship between ADHD and (1) job shifting, (2) relational instability, and (3) residential moves throughout follow-up. The analyses were stratified by age groups (18–29, 30–39, and 40–52 years at the start of follow-up) and sex (males, females). We first examined crude associations controlling for birth year and parent educational level. We then adjusted for SUD, BPD and criminal convictions. Effects of age and sex were tested in separate models. First, an interaction between ADHD and age group was included to determine whether the associations between ADHD and our outcomes were different among different age groups. Second, a separate test of interactions between ADHD and sex were included to assess if there were differences between males and females within each outcome. Finally, we tested a 3-way interaction between age x sex x ADHD to assess whether the sex-ADHD interaction varied in different age groups. Estimates are presented as incidence rate ratio (IRR) with 95% confidence intervals (CI).

To address the issue that some individuals are potentially not able to work and move around as much as other people, we did a sensitivity analysis where we excluded individuals with (1) severe intellectual disability, (2) an incarceration in prison longer than 2 years, (3) an annual/and or work-related income at baseline < 41,800 SEK/4,461 EUR, (4) disability pension at baseline, and (5) long term sick leave at baseline (> 183 days). Data management and statistical analyses were performed using SAS software version 9.4 (SAS Institute, Cary, NC, USA) and Stata 15 [40].

## Results

The demographic characteristics of the study population are presented in Table 1. Of the 3,448,400 individuals in this Swedish nationwide population 17,088 men (0.91%) and 13,993 women (0.84%) had an ADHD-diagnosis at some point during the follow up time. The prevalence of SUD (40%) and BPD (9.8%) were higher among individuals with ADHD compared to individuals without ADHD (SUD = 4.6%; BPD = 0.5%). Parental income was lower among individuals with ADHD. Table S1 presents the average number of job shifting, relational instability, and residential moves in the study population.

**Table 1:** Demographic characteristics of the study population

	Total population	Without ADHD	With ADHD	p-value^2^
	N = 3,448,440^1^	N = 3,417,559^1^	N = 31,081^1^	
Age	34 (26,43)	34 (26,43)	28 (22,35)	< 0.001
Sex				
Males	1,770,673 (51%)	1,735,585 (51%)	17,088 (55%)	
Females	1,677,767 (49%)	1,663,774 (49%)	13,993 (45%)	
SUD	168,994 (4,9%)	156,498 (4,6%)	12,496 (40%)	< 0.001
BPD	21,019 (0,6%)	17,978 (0,5%)	3,041 (9, 8%)	< 0.001
Income parents	2,612 (1,977, 3,369)	2,619 (1,990, 3,376)	1,577 (1,204, 2,289)	< 0.001
Education level parents				< 0.001
Primary school	382,373 (12%)	374,545 (12%)	7,828 (26%)	
Secondary school	1,750,282 (55%)	1,733,184 (55%)	17,098 (57%)	
University	1,031,867 (33%)	1,026,672 (33%)	5, 195 (17%)	

ADHD = Attention-Deficit/Hyperactivity-Disorder; SUD = Substance Use Disorder; BPD = Borderline Personality Disorder; ^1^ Median (IQR); n (%), ^2^ Wilcoxon rank sum test; Pearson´s Chi-squared test.

### Associations between ADHD and real-life instability from young to middle adulthood

Adults with ADHD had an increased rate of residential moves (IRR = 2.35 [95% CI, 2.32–2.37], p < 0.001), relational instability (IRR = 1.07 [95% CI, 1.06–1.08], p < 0.001) and job shifting (IRR = 1.03 [95% CI, 1.02–1.04], p < 0.001) compared to individuals without ADHD. As can be seen from the age-stratified results in Table 2, these associations tended to increase with increasing age. All associations were significantly stronger in the older age groups (30–39 years and 40–52 years at start of follow up) compared to the youngest group (18–29 years at start of follow up), see Table 3. The older group also differed significantly from the middle age group on all of our outcomes (Residential moves IRR = 1.50 [1.47–1.54], p < 0.001, Relational instability IRR = 1.11[1.08–1.14], p < 0.001, Job shifting IRR = 1.09 [1.06–1.12], p < 0.001).

**Table 2:** Associations between ADHD and job shifting, children with different partners and residential moves

	Age 18–29		Age 30–39		Age 40–52	
	IRR (95% CI)		IRR (95% CI)		IRR (95% CI)	
	Crude	Adjusted	Crude	Adjusted	Crude	Adjusted
Job shifting Males	0.99 (0.97–1.01)	0.96 (0.94–0.98)	1.12* (1.08–1.16)	1.05* (1.02–1.09)	1.13* (1.07–1.21)	1.10* (1.03–1.17)
Job shifting Females	0.97 (0.95–0.99)	0.96 (0.94–0.98)	1.10* (1.06–1.14)	1.04 (1.01–1.09)	1.23* (1.13–1.34)	1.18* (1.08–1.28)
Children with different partners Males	0.93 (0.91–0.96)	0.95 (0.93–0.97)	1.09* (1.07–1.12)	1.09* (1.06–1.11)	1.09* (1.05–1.16)	1.05* (1.02–1.09)
Children with different partners Females	1.04 (1.01–1.06)	1.04 (1.01–1.06)	1.22* (1.19–1.24)	1.17* (1.14–1.1.19)	1.18* (1.14–1.22)	1.14* (1.10–1.18)
Residential moves Males	1.48* (1.45–1.49)	1.25* (1.23–1.27)	2.50* (2.43–2.57)	1.62* (1.58–1.67)	2.60* (2.46–2.75)	1.69* (1.60–1.78)
Residential moves Females	1.41* (1.39–1.43)	1.23* (1.21–1.25)	2.14* (2.08–2.21)	1.56* (1.51–1.61)	2.28* (2.14–2.42)	1.68* (1.58–1.79)

**Note**: IRR = incidence ratio; ADHD = Attention-Deficit/Hyperactivity-Disorder; Crude = Adjusted for birth year and maternal- and parental education; Adjusted = Adjusted for birth-year, maternal- and paternal education, BPD, SUD and Criminal Convictions. *=Significant at p < 0.001

Males and females with ADHD in the youngest group did not differ significantly from controls without ADHD on job shifting and relational instability (Table 2). They did however have a higher rate of residential moves (IRR Males = 1.48 [95% CI, 1.45–1.49], p < 0.001; IRR Females = 1.41 [95% CI, 1.39–1.43], p < 0.001) see Table 2. The strongest associations between ADHD and real-life instability were found in the oldest group. Males and females (41–52 years) with ADHD had a significantly higher rate of job shifting (IRR Males = 1.13 [95% CI, 1.07–1.21], p < 0.001; IRR Females = 1.23 [95% CI, 1.13–1.34], p < 0.001), relational instability (IRR Males = 1.09 [95% CI, 1.05–1.16], p < 0.000; IRR Females = 1.18 [95% CI, 1.14–1.22], p < 0.001), and residential moves (IRR Males = 2.60 [95% CI, 2.46–2.75], p < 0.001; IRR Females = 2.28 [95% CI, 2.14–2.42], p < 0.001) compared to individuals in the same age without ADHD (Table 2). These crude associations were adjusted for birth year and maternal- and paternal educational level. The effect size attenuated for many of the observed associations after adjustment for BPD, SUD and criminal convictions, but all associations remained statistically significant. see Table 2.

**Table 3:** Interactions between age and job shifting, children with different partners and residential moves

	Age 30–39		Age 40–52	
	IRR (95% CI)		IRR (95% CI)	
	Adjusted	P	Adjusted	P
Job shifting	1.13 (1.09–1.16)	< 0.001	1.20 (1.14–1.27)	< 0.001
Children with different partners	1.18 (1.16–1.21)	< 0.001	1.14 (1.11–1.18)	< 0.001
Residential Moves	1.56 (1.53–1.60)	< 0.001	1.68 (1.61–1.75)	< 0.001

**Note**: Age 18–29 as reference group, IRR = 1. IRR = incidence ratio; ADHD = Attention-Deficit/Hyperactivity-Disorder; Adjusted = Adjusted for birth-year, maternal- and paternal education, BPD, SUD and Criminal Convictions

### Sex differences on the association between ADHD and real-life instability

As can be seen in Table 4, there was no significant difference between males and females with ADHD in the youngest age group (18–29 years) on job shifting (p = 0.936). However, compared with men, women with ADHD in the youngest age group had a significantly higher rate of relational instability (IRR = 1.18 [95% CI, 1.14–1.21], p < 0.001) and residential moves (IRR = 1.04 [95% CI, 1.02–1.07], p < 0.001) compared to men with ADHD. Women with ADHD in the two older age groups (30–39 years and 40–52 years) also had a significantly increased rate of relational instability (IRR 30–39 years = 1.15 [95% CI, 1.11–1.18], p < 0.001; IRR 41–52 years = 1.11 [95% CI, 1.06–1.17], p < 0.001) compared to men with ADHD, see Table 4. When examining how the moderating effect of sex on the relationship between ADHD and our outcomes could vary among different ages (i.e., sex × ADHD × age interaction), we found that this interaction did not vary at different age groups for job shifting (30–40, p = 0.743; >40 p = 0.084). For total moves, we found a significant 3-way interaction for the oldest age group (30–40 p = 0.726; >40 p < 0.001), and lastly, for relational instability, we saw a significant 3-way interaction for the age group 30–40 (p = 0.022), but not for the > 40 group (p = 0.173).

**Table 4:** Sex influences on the association between ADHD and job shifting, children with different partners and residential moves

	Age 18–29		Age 30–39		Age 40–52	
	IRR (95% CI)		IRR (95% CI)		IRR (95% CI)	
	Adjusted	P	Adjusted	P	Adjusted	P
Job shifting	1.00 (0.97–1.03)	0.936	1.00 (0.95–1.05)	0.984	1.12 (1.01–1.25)	0.033
Children with different partners	1.18 (1.14–1.21)	< 0.001	1.15 (1.11–1.18)	< 0.001	1.11 (1.06–1.17)	< 0.001
Residential Moves	1.04 (1.02–1.07)	< 0.001	0.98 (0.94–1.02)	0.307	0.99 (0.91–1.07)	0.803

**Note**: Males as reference. IRR = incidence ratio; ADHD = Attention-Deficit/Hyperactivity-Disorder; Crude = Adjusted for birth year and maternal- and parental education; Adjusted = Adjusted for birth-year, maternal- and paternal education, BPD, SUD and Criminal Convictions

### Sensitivity analyses

All associations remained similar after excluding individuals; with severe intellectual disability, who were convicted for any crime more than two years, and/or were not in paid work, had disability pension, had long-term sick-leave at baseline, see Table S2.

## Discussion

To our knowledge, this is the first study examining associations between ADHD and register-based indicators of real-life instability, including job shifting, relational instability, and residential moves. Our primary finding was that both men and women with a diagnosis of ADHD presents with an increased risk of real-life instability in different domains, in particular beyond young adulthood. These findings shed important light on the real-life functioning of individuals with ADHD across the lifespan. Such information is critical given that recent research indicate that a large number of adults show elevated levels of ADHD symptoms, [41], but a detailed understanding of real-life functioning has until now been lacking in this age group. An increased awareness of real-life instability in ADHD across the lifespan may help reduce problems related to under-diagnosis and failures to provide adequate support for relevant real-life functional impairments in ADHD beyond young adulthood. Increased awareness of these risks are important for individuals with a diagnosis ADHD, their families and health care professionals because these factors are in themselves associated with negative outcomes in life, such as lower income, worse living conditions, and potentially harmful effects on children [11, 42].

Our findings of an increased risk of job shifting, relationship instability, and residential moves in adults with ADHD are broadly consistent with previous studies on social and occupational outcomes in adults with ADHD [12, 13, 16], but we were able to extend the available knowledge-base in three important ways.

First, the large sample size and long timescale allowed us to study associations between ADHD and real-life instability over 14 years in people aged 18 to 52 years at the start of follow-up. This allowed us to study real-life instability across a large part of the adult lifespan covering younger to middle aged adults. Our findings indicate that associations with real-life instability were more pronounced in middle aged adults with ADHD compared to younger adults with ADHD. The young adults with ADHD (18–29 years at start of follow up) had an 0–25% increased risk of real-life instability compared to individuals without ADHD. In contrast, the older groups with ADHD (30–39 and 40–52 years at start of follow up) had an 4–62% and 5–69% increased risk of real-life instability respectively compared to individuals without ADHD. One potential explanation for this pattern is that real-life instability is more normative in young adults, even in those without a diagnosis of ADHD. However, individuals with ADHD in the youngest age group (18–29 years) tended to make more residential moves compared to individuals without ADHD in the same age.

Second, our large sample size allowed us to present results separately for males and females. Overall, a similar pattern emerged across sex, suggesting that both males and females with ADHD show an increased risk of real-life instability. Our findings may, however, suggest that women with ADHD have a more pronounced risk for relationship instability compared to men with ADHD. Higher rates of relationship instability in females with ADHD is in line with findings from a recent review of females with a diagnosis of ADHD [15].

Third, we found that psychiatric comorbidity and severe behavioral problems (e.g., criminality) influence the risk of real-life instability in ADHD. These findings are consistent with a large body of research indicating that co-occurring psychiatric and behavioral problems have an important role for functional outcomes in ADHD, such as mortality, suicidal behavior, and educational level [43–46]. Even though our results demonstrated that associations attenuated after adjustment for these covariates, the associations between ADHD and different outcomes remained statistically significant but were quite small (IRR´s ranged from 1.05 to 1.69). Small effects were expected considering that our indicators of real-life instability are crude and multifactorial in nature. However, our findings are consistent with the hypothesis that core characteristics of ADHD, such as inattention, hyperactivity, and impulsivity manifest in real-world instability. Our findings point to the need of raising the awareness of real-life instability in adults with ADHD, from young to older adulthood.

### Limitations

Register-based studies capture only treatment seeking individuals. The ADHD-prevalence of 0.91% in our population-based sample is in line with other register-based studies on adult ADHD, but lower when compared with the 2.5% found in studies were all people with ADHD are identified - both treatment seeking individuals with earlier diagnoses and also not previously diagnosed individuals [47, 48]. We have a possible higher under-identification of ADHD in the older ages in our study. It should also be noted that the registers capture the more severe cases with ADHD, which limit the generalizability of our findings. Given the nature of the available register-data we were unable to explore potential differences between DSM ADHD subtypes (i.e., combined presentation, predominantly inattentive presentation and predominantly hyperactive/impulsive presentation). Thus, we cannot rule out potential differences between the ADHD presentations.

Using the number of children with different partners as a measure of relational instability is a crude measure. Also, there is a possibility that outcome misclassification is more pronounced in fathers than in mothers for our indicator of relational instability. This could potentially explain why we only see an association in females but not in males. However, the frequency of misattributed paternity in Sweden is low [49]. Using job shifting as a measure of real-life instability in young adulthood (18–29 years at start of follow up in our cohort) is not ideal since a lot of people in those ages are studying and a relatively high degree of job shifting is also normative. This can be a possible explanation to why we did not see an increased risk of job shifting in individuals with ADHD in younger adulthood. We used a broad definition of young adulthood (i.e., 18–29 years). While our selected age range for this group increased the possibility for variation in our indicators of instability we might also have introduced heterogeneity, which might limit the generalizability of our results to more narrow definitions of young adulthood (i.e., 18–25 years).

Overall, our indicators of instability have the advantage of being concrete reflections of real-life functioning, which could be useful in clinical settings to guide discussions around the treatment plan. On the other hand, our crude real-life indicators may miss important nuances of instability.

Our covariates SUD and criminal convictions are robust real-life measures but are indicators of the most severe end of externalizing problem and is probably lacking sensitivity as indicators of more moderate forms of externalizing problems. Since BPD is underdiagnosed in clinical practice and therefore also in the registers, this covariate probably lacks sensitivity as well [50]. Our selected covariates only adjust for measured confounding. It is possible that the associations would have further attenuated if we also adjusted for unmeasured confounding. Lastly, using job shifting as a measure of real-life instability in young adulthood (18–29 years at start of follow up in our cohort) is not ideal since a lot of people in those ages are studying and a relatively high degree of job shifting is also normative. This can be a possible explanation to why we did not see an increased risk of job shifting in individuals with ADHD in younger adulthood.

## Conclusions

Both men and women with a diagnosis of ADHD present with an increased risk of real-life instability in different domains; a behavioral pattern that is not limited to childhood or adolescence but also exist well into adulthood. Clearly, it is important to have a lifespan perspective on ADHD for individuals, relatives and the health care sector.

### Electronic supplementary material

Below is the link to the electronic supplementary material.

Supplementary Material 1 Table S1 and Table S2

## Data Availability

The data that support the findings of this study are available from Statistics Sweden but restrictions apply to the availability of these data, which were used under license for the current study, and so are not publicly available. Data are however available from the authors (henrik.larsson@oru.se) upon reasonable request and with permission of Statistics Sweden.
