# Supplementary material for: Real-life instability in ADHD from young to middle adulthood: a nationwide register-based study of social and occupational problems
Source: BMC Psychiatry. 2023 May 12;23:336. doi: 10.1186/s12888-023-04713-z (PMC10176742; doi:10.1186/s12888-023-04713-z)
Supplement: Supplementary file 1 — Supplementary Material 1 Table S1 and Table S2 [file 12888_2023_4713_MOESM1_ESM.pdf]

Supplementary information

Table S1. Number of job shifts, children with different partners, and residential moves in the study population (n=3,448,440) from 2000-2012.

	Age 18-29		Age 30-39		Age 40-52	
	Mean (SD)		Mean (SD)		Mean (SD)	
	With ADHD	Without ADHD	With ADHD	Without ADHD	With ADHD	Without ADHD
Job shifts Males	2.17 (1.94)	2.49 (1.84)	2.16 (1.96)	2.04 (1.76)	1.61 (1.78)	1.33 (1.52)
Job shifts Females	2.15 (1.97)	2.53 (1.85)	1.86 (1.80)	1.84 (1.72)	1.34 (1.65)	1.07 (1.42)
Children with different partners Males	0.49 (0.66)	0.48 (0.55)	0.75 (0.77)	0.74 (0.57)	0.97 (0.81)	0.86 (0.61)
Children with different partners Females	0.71 (0.77)	0.66 (0.57)	1.04 (0.86)	0.87 (0.56)	1.21 (0.84)	0.95 (0.57)
Residential moves Males	5.34 (3.71)	4.36 (2.72)	4.42 (3.54)	2.72 (2.34)	2.95 (3.14)	1.01 (1.57)
Residential moves Females	6.10 (3.71)	5.04 (2.88)	4.22 (3.42)	2.69 (2.36)	2.64 (2.90)	1.03 (1.55)

Note: ADHD=Attention-Deficit/Hyperactivity-Disorder; SD=Standard Deviation.

Table S2. Sensitivity analysis. Associations between ADHD and job shifting, children with different partners and residential moves. *

	Age 18-29		Age 30-40		Age 40-52	
	IRR (95 % CI)		IRR (95 % CI)		IRR (95 % CI)	
	Crude	Adjusted	Crude	Adjusted	Crude	Adjusted
Job shifting Males	1.06* (1.04-1.08)	1.02 (0.99-1.04)	1.18* (1.14-1.22)	1.10* (1.06-1.14)	1.19* (1.11-1.27)	1.14* (1.06-1.22)
Job shifting Females	1.01 (0.98-1.04)	0.99 (0.96-1.02)	1.13 (1.09-1.18)	1.07 (1.02-1.11)	1.30* (1.18-1.43)	1.23* (1.12-1.35)
Children with different partners Males	0.97 (0.94-1.00)	0.99 (0.96-1.01)	1.13* (1.11-1.16)	1.11* (1.08-1.14)	1.12* (1.08-1.16)	1.08* (1.04-1.12)
Children with different partners Females	1.08* (1.05-1.11)	1.07* (1.05-1.09)	1.23* (1.20-1.26)	1.17* (1.14-1.20)	1.16* (1.12-1.21)	1.12* (1.08-1.17)
Residential moves Males	1.53* (1.51-1.56)	1.30* (1.28-1.33)	2.42* (2.34-2.50)	1.65* (1.60-1.71)	2.44* (2.27-2.62)	1.69* (1.57-1.81)
Residential moves Females	1.46* (1.44-1.49)	1.28* (1.25-1.30)	2.15* (2.08-2.23)	1.59* (1.53-1.65)	2.27* (2.10-2.45)	1.79* (1.65-1.95)

Note: IRR=incidence ratio; ADHD= Attention-Deficit/Hyperactivity-Disorder; Crude=Adjusted for sex and birth year; Adjusted=Adjusted for sex, birth-year, maternal- and paternal education, Borderline Personality Disorder, Substance Use Disorder, Criminal Convictions.
*= Excluding Those who had severe intellectual disability; Were convicted for any crime for more than 2 years; Who were not in paid work (having annual work-related income < 41,800 SEK/4,461 EUR); Who had disability pension at baseline; Who had long-term sick leave at baseline | >183 days.
